# 

**Published:** 1886-05

**Authors:** 


### NOTICE TO DELINQUENT SUBSCRIBERS.

One more issue of this journal will complete Vol. 1. Those who have received the JOURNAL regularly, and who have not paid the subscription, are respectfully informed that we will *draw a five days' draft on each*, accompanied by a receipted bill for the years' subscription, ending with the June number.

We are compelled to take this course; it being impossible to write to each subscriber; and we feel assured that the gentlemen who have *accepted* the JOURNAL regularly, although they may not have authorized the sending of it, and who have had the benefit of a year's subscription, only need to be reminded of the fact that the small amount of $2 *is expected of each*, to cheerfully pay it, we believe. We hope none will feel offended; and we say now to those who have received the JOURNAL, under circumstances stated, if there are any who do not feel a moral obligation to pay the draft, they need not do so.

After this, all who have not paid, will be dropped.

We earnestly ask the support of the profession in our laudable work, and we most heartily thank the large number of physicians who have so cheerfully and so promptly aided us, and who have thereby made the JOURNAL a success, of which the profession of Texas is truly proud. When the June number is out, and the volume is completed, we trust our paid subscribers will promptly renew their subscriptions, if they would keep the JOURNAL going.

